# Late onset mixed delirium after reconstructive breast operation revealed pituitary macroadenoma: a rare case report

**DOI:** 10.1192/j.eurpsy.2025.1236

**Published:** 2025-08-26

**Authors:** I. Grammatikopoulos, A. Tarar, M. Ijaz, M. McDonald, B. O’Leary, J. Chandrakanth

**Affiliations:** 1 DEPARTMENT OF PSYCHIATRY, UNIVERSITY HOSPITAL WATERFORD, WATERFORD, Ireland

## Abstract

**Introduction:**

Delirium has been considered a reflection of diffuse cerebral metabolic insufficiency, and the pathophysiology of underlying delirium is quite diverse.

**Objectives:**

We present a rare case of mixed delirium with shifting activity level between the hyperactive and hypoactive delirium types as the only manifestation in a 42-year-old woman presented with psychotic symptoms without any prior psychiatric history, one month after a reconstructive breast cancer operation.

**Methods:**

To provide an overview, describe the clinical features and differential diagnosis, as psychiatric conditions are among the most difficult to differentiate from delirium, and finally review the clinical management.

**Results:**

Presentation of a 42-year-old female with a history of right breast cancer with triple negative B5b invasive carcinoma, twice operated after local recurrence (first operation 4 years ago), and a second reconstructive operation one month ago, with no past psychiatric history, who presented with symptoms of mixed delirium. During the clinical and laboratorial investigation, exams revealed hyperprolactinemia. The patient showed no other clinical signs or symptoms compatible with adrenal insufficiency and displayed normal ACTH and serum cortisol concentrations. Brain magnetic resonance imaging revealed pituitary macroadenoma without any brain metastasis. The patient was treated with antipsychotics and not corticosteroids, resulting in rapid remission of the psychotic symptoms.

**Conclusions:**

Clouded sensorium and behavioral dysregulation in delirium can be easily mistaken as thought and behavioral disorganization in acute psychotic episodes, and detailed evaluation of precipitating factors is required to differentiate delirium.

**Disclosure of Interest:**

None Declared

